# NfκBin: a machine learning based method for screening TNF-α induced NF-κB inhibitors

**DOI:** 10.3389/fbinf.2025.1573744

**Published:** 2025-07-17

**Authors:** Shipra Jain, Ritu Tomer, Sumeet Patiyal, Gajendra P. S. Raghava

**Affiliations:** ^1^ Department of Computational Biology, Indraprastha Institute of Information Technology, New Delhi, India; ^2^ Cancer and Data Science Laboratory (CDSL), National Cancer Institute, National Institutes of Health, Bethesda, MD, United States

**Keywords:** NF-κB, nuclear factor kappa B, machine learning, chemical descriptors, high-throughput screening, inhibitor prediction tool

## Abstract

**Introduction:**

Nuclear Factor kappa B (NF-κB) is a transcription factor whose upregulation is associated in chronic inflammatory diseases, including rheumatoid arthritis, inflammatory bowel disease, and asthma. In order to develop therapeutic strategies targeting NF-κB-related diseases, we developed a computational approach to predict drugs capable of inhibiting TNF-α induced NF-κB signaling pathways.

**Method:**

We utilized a dataset comprising 1,149 inhibitors and 1,332 non-inhibitors retrieved from PubChem. Chemical descriptors were computed using the PaDEL software, and relevant features were selected using advanced feature selection techniques.

**Result:**

Initially, machine learning models were constructed using 2D descriptors, 3D descriptors, and molecular fingerprints, achieving maximum AUC values of 0.66, 0.56, and 0.66, respectively. To improve feature selection, we applied univariate analysis and SVC-L1 regularization to identify features that can effectively differentiate inhibitors from non-inhibitors. Using these selected features, we developed machine learning models, our support vector classifier achieved a highest AUC of 0.75 on the validation dataset.

**Discussion:**

Finally, this best-performing model was employed to screen FDA-approved drugs for potential NF-κB inhibitors. Notably, most of the predicted inhibitors corresponded to drugs previously identified as inhibitors in experimental studies, underscoring the model’s predictive reliability. Our best-performing models have been integrated into a standalone software and web server, NfκBin. (https://webs.iiitd.edu.in/raghava/nfkbin/).

## Highlights

• NF-κB signaling pathway plays crucial role in many diseases like rheumatoid arthritis.
• NF-κB signaling pathway is drug target for arthritis, bowel disease, asthma etc.
• A method for classification of TNF-α induced NF-κB inhibitors and non-inhibitors.
• Application machine learning techniques for predicting inhibitors.
• A web server NfκBin for predicting, designing and screening inhibitors.

## 1 Introduction

**FIGURE 1:**
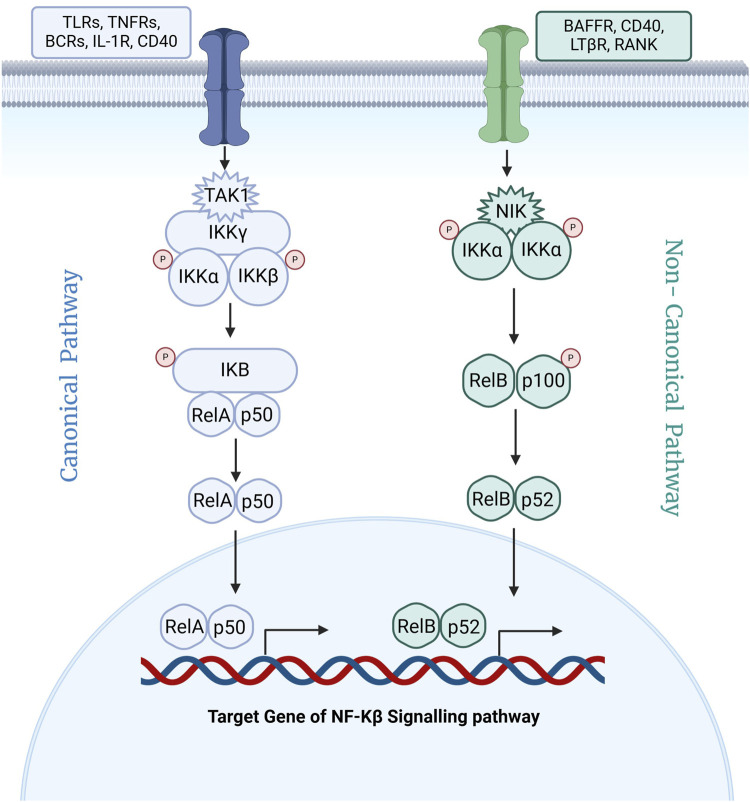
Schematic representation of canonical and non-canonical signaling pathway for activation of Nf-κB.

Nuclear factor kappa B (NF-κB) is a pivotal transcription factor that regulates genes critical for immune and inflammatory responses (Lawrence, 2009). Since its discovery in 1986, NF-κB has been identified as central to the body’s defense mechanisms (Hayden and Ghosh, 2008). It is activated by receptors such as Toll-like receptors (TLRs), which detect microbial components and trigger inflammatory in response to harmful stimuli like pathogens, damaged cells, and irritants (Lim and Staudt, 2013; Tak and Firestein, 2001; Li and Verma, 2002). As depicted in Figure 1, NF-κB activation occurs via two main pathways: canonical and non-canonical (Bonizzi and Karin, 2004; Hayden and Ghosh, 2014; Wu and Zhou, 2010; Hoesel and Schmid, 2013; Baud and Karin, 2009). The canonical pathway, triggered by signals such as TNF-α and IL-1, involves the phosphorylation and degradation of IκB, allowing NF-κB to translocate into the nucleus and initiate transcription of genes related to inflammation and immunity (Lawrence, 2009; Schütze et al., 1995; Hayden and Ghosh, 2004). The non-canonical pathway, activated by receptors like CD40 and BAFF, relies on NIK-mediated processing of p100 into its active form (p52), which pairs with RelB to promote immune system development and adaptive immune responses (Hayden and Ghosh, 2014).

Dysregulated NF-κB signaling is implicated in numerous diseases, including chronic inflammatory conditions (e.g., Crohn’s disease, asthma, and psoriasis), autoimmune disorders (e.g., SLE and multiple sclerosis), and cancers (e.g., breast, lung, and colorectal cancers) (Hoesel and Schmid, 2013; Zinatizadeh et al., 2020; Lee et al., 2007). Persistent activation contributes to excessive inflammation, tissue damage, and tumor progression by promoting cell survival, proliferation, and resistance to apoptosis (Liu et al., 2017; Yamamoto and Gaynor, 2001). Moreover, it fosters tumor invasion, metastasis, and angiogenesis by creating a pro-tumor microenvironment (Rasmi et al., 2020; Tang et al., 2017; Pikarsky et al., 2004; Dong et al., 2015; Karin, 2006; Zhao et al., 2021).

Due to its central role in diverse diseases, NF-κB is a promising therapeutic target (Wu and Zhou, 2010; Baldwin, 1996). Existing inhibitors range from small molecules to natural compounds and peptides, targeting various stages of NF-κB signaling (Yamamoto and Gaynor, 2001). However, traditional drug development methods are expensive and time-consuming. Computational approaches for high-throughput screening of chemical libraries to identify NF-κB inhibitors are urgently needed. Among various NF-κB activation pathways, the TNF-α-induced canonical pathway is one of the most extensively studied and clinically relevant, making it a suitable focus for targeted inhibitor discovery. Targeting this specific axis allows for the identification of compounds capable of modulating early upstream events in NF-κB signaling, offering broad therapeutic potential across inflammation-related diseases and cancer. In present study, we introduce “NFκBIn,” an *in silico* tool for predicting TNF-α-induced NF-κB inhibitors based on experimentally validated compounds. This tool addresses the gap in computational resources for efficient and precise inhibitor prediction.

## 2 Methods

### 2.1 Dataset collection

In this study, we extracted the TNF-α induced NF-κB inhibitors and non-inhibitors from the PubChem repository (Wang et al., 2012). We filtered all the assays in the aforementioned repository using keywords “((TNF AND NF-κB) inhibitors)”. This search resulted in a total of 90 PubChem bioassays, which was further manually refined based on the number of inhibitors per assay. After rigorous screening we selected a high throughput bioassay AID 1852 (https://pubchem.ncbi.nlm.nih.gov/bioassay/1852) as the data source for our study. This high throughput assay is designed for identification of hits specific to tumor necrosis factor alpha (TNF-α), a canonical NF-κB inducer, and its modulated pathways. HEK-293-T NF-κB-Luc cells were seeded at 6,000 cells/well in 1,536-well plates with 0.62% DMSO and treated with 10 nL of 2 mM compounds or DMSO controls using a pintool. After stimulation with 0.25 ng/mL TNF-α and overnight incubation, luminescence was measured using SteadyGlo and a Perkin-Elmer Viewlux reader. Tiered Activity Scoring System developed by Sanford-Burnham Center for Chemical Genomics (SBCCG), was deployed and the compounds showing more than 50% activity in the assay were classified as active. Using this assay, we downloaded a total of 2,481 compounds in which 1,332 were non-inhibitors and 1,149 were reported as NF-κB inhibitors, using this bioassay.

For the drug repurposing case study, we retrieved 2,616 FDA-approved small molecules from the DrugBank database. Of these, 2,577 compounds with available SMILES representations were used for descriptor generation and prediction using the “Predict” Module of NFκBIn webserver.

**FIGURE 2:**
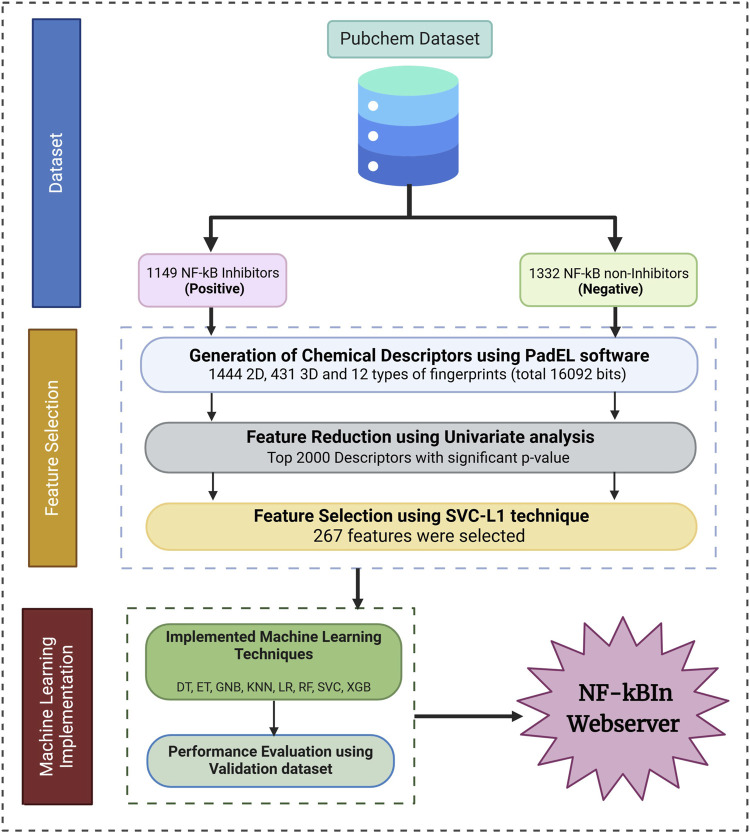
Overall architecture depicting workflow of Nf-κBIn tool.

The detailed schematic representation of the proposed tool is illustrated in Figure 2.

### 2.2 Dataset preprocessing

In this study, we followed the best practices of machine learning algorithms and divided our total compound dataset in 80:20 ratio. Where, 80% of data (i.e. 936 inhibitors and 1048 non-inhibitors) was flagged as training data and were utilized to develop machine learning models and remaining 20% data (i.e. 213 inhibitors and 284 non-inhibitors) was used as independent validation set for machine learning model performance evaluation. These types of standard protocols were reported in previous studies from literature (Dhanda et al., 2013; Chauhan et al., 2014; Sharma et al., 2021).

### 2.3 Molecular descriptors and fingerprints of compounds

Molecular descriptors and fingerprints are the mathematical representation of chemical compounds that captures vital information about them (Yap, 2011; Yang et al., 2022; Dhall et al., 2021). The descriptors are key features extracted to represent chemical compounds in computational chemistry and drug discovery (Boldini et al., 2024). They help in predicting the biological activity, physicochemical properties, and toxicity of compounds. In this study, we deployed PaDEL software (Yap, 2011) for calculation of molecular and fingerprint descriptors of NF-κB inhibitors and non-inhibitors downloaded in SMILES format. This software calculated 1,875 descriptors including 1444 1D, 2D; 431 3D and 12 types of fingerprints (total 16,092 bits). These 17967 2-D, 3-D, and fingerprint (FP) descriptors were further screened to develop machine learning algorithms.

### 2.4 Descriptor features preprocessing

The 17,967 generated descriptors exhibited varying range values. To normalize them, we applied the Standard Scaler from the Scikit-learn package, which operates using the z-score algorithm (Dhall et al., 2021). Post this step, we discarded the descriptors with more than 80% null values. After this we were left with 1107 2D, 431 3D and 9324 FP descriptors, making a total of 10,862 descriptors/features for the dataset.

### 2.5 Significant descriptor selection and ranking

In order to develop a robust prediction model with higher accuracy, we need to select the most significant descriptors generated from PaDEL software. Thus, ranking and selecting the significant descriptors from the 10,862 descriptors set is an important step. In this study we incorporated two approaches to select and rank relevant descriptors, i.e., using correlation analysis and univariate analysis.

#### 2.5.1 Correlation based Descriptor selection

In this approach, we deployed the Variance Threshold package of Scikit (sklearn.feature_selection) to remove the low-variance features from 10,862 descriptor set. After eliminating low variance features, we were left with 6084 descriptors comprising of 786 2D, 169 3D and 5129 FP features (Refer Supplementary Table S1 in Supplementary Material). We applied a Pearson correlation-based feature selection method to remove highly correlated features, using a cutoff value of 0.6 (Dhall et al., 2021). Post this step, we were left with 102 2D, 3 3D and 2260 FP descriptors making a total of 2365 descriptors (See Supplementary Table S2 in Supplementary Material). In order to further reduce the dimensionality of the descriptor matrix, we applied SVC-L1 based feature selection method to screen relevant feature set. The support vector classifier (SVC) with linear kernel and L1 regularization is the foundation of this approach (Kamkar et al., 2016). Using SVC-L1 feature selection method we selected, 32 2D, 3 3D and 348 FP feature set (Refer Supplementary Table S3 in Supplementary Material for detailed list). Using these descriptors, we developed 2D, 3D, FP and ensemble-based machine learning models to screen NF-κB inhibitors.

#### 2.5.2 Univariate analysis-based Descriptor selection

In this approach, a statistical method i.e., univariate analysis using 2-tailed independent Student’s t-test was executed based on the mean value of descriptors of both groups to extract the important descriptors from the 10,862 descriptors pool. Using this approach, we ranked the descriptors based on the significant p-value obtained. We selected top 2000 descriptors and applied SVC-L1 and RFE based feature extraction methods over them. Recursive Feature Elimination (RFE) is a feature selection approach that works by recursively eliminating the least important features, this process continues until the desired number of features is reached (Chen and Jeong, 2007). Applying these, we selected 266 descriptors from SVC-L1 method and the top 50 descriptors from RFE feature selection technique for machine learning model development.

### 2.6 Cross validation techniques

In order to achieve an unbiased prediction model, we incorporated standard five-fold cross validation techniques, to build our machine learning models (Sharma et al., 2021; Dhall et al., 2021; Jain et al., 2022). In this technique, we divided our 80% training dataset into five sets of data with similar size. Out of these five sets, four sets were used to train the machine learning model and one set was used for testing the machine learning model performance. This process was repeated five times, to make sure that each fold is used once for testing the model. We fine-tuned the machine learning models parameters for achieving best performance on the test dataset. Finally, the average performance was computed using five test folds performance.

### 2.7 Machine learning models

In this study we have applied various machine learning algorithms to develop prediction models for screening of NF-κB inhibitors and non-inhibitors with higher accuracy. We implemented Random Forest (RF), Decision Tree (DT), K-nearest neighbour (KNN), Support Vector Classifier (SVC), and eXtreme Gradient Boosting (XGB) to develop classification models. These machine learning algorithms were deployed using the Scikit-learn package (Pedregosa et al., 2012).

### 2.8 Performance evaluation

We have evaluated our machine learning model performance over 20% independent validation dataset. We recorded both threshold-dependent and independent parameters for evaluating our model’s performance. As explained in Equations 1–4 sensitivity (Sens), specificity (Spec), accuracy (Acc), and Matthew’s correlation coefficient (MCC) respectively, were recorded as threshold-dependent parameters and the area under the receiver operating characteristic curve (AUC), as the threshold-independent parameter (Jain et al., 2022; Sharma et al., 2022).

$$Sensitivity=\frac{TP}{TP+FN}*\;100$$ (1)

$$Specificity=\frac{TN}{TN+FP}\;*100$$ (2)

$$Accuracy=\frac{TP+TN}{TP+FP+TN+FN}*100$$ (3)

$$MCC=\frac{\left(TP*TN\right)-\left(FP*FN\right)}{\sqrt{\left(TP+FP\right)\left(TP+FN\right)\left(TN+FP\right)\left(TN+FN\right)}}$$ (4)

Where, FP is false positive, FN is false negative, TP is true positive and TN is true negative.

## 3 Results

### 3.1 Functional group analysis

**FIGURE 3:**
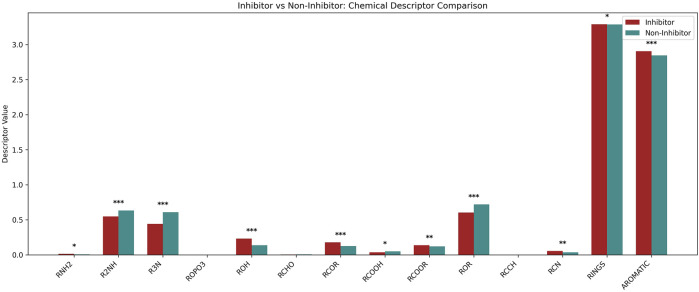
Representation of functional group analysis for Nf-κB pathway inhibitors and non-inhibitors using ChemmineR package.

In order to get the deeper insights of relevance of functional groups present in NF-κB signaling pathway inhibitors and non-inhibitors. We used ChemmineR package to detect and the frequency of functional groups in both NF-κB inhibitors and non-inhibitors chemical compounds (Cao et al., 2008). Using this approach, we observed the occurrence of Primary (RNH2), Secondary (R2NH), Tertiary (R3N) Amines, Phosphates attached to alkyl groups (ROPO3), Alcohol (ROH), Aldehyde (RCHO), Ketone (RCOR), Carboxylic Acid (RCOOH), Ester (RCOOR), Ether (ROR), Alkyne (RCCH), Nitrile (RCN), Rings and Aromatic groups for our positive and negative dataset. The frequency of these functional groups is depicted in Figure 3. Statistical significance between both groups was assessed using an unpaired two-tailed t-test, with annotations as follows: ***p < 0.001, **p < 0.01, *p < 0.05; non-significant comparisons (p ≥ 0.05) were not labeled.

The comparative analysis of chemical descriptors revealed distinct structural preferences between inhibitors and non-inhibitors. Descriptors such as RNH2, ROH, RCOR, and AROMATIC were significantly enriched in inhibitors, with ROH, RCOR, and AROMATIC showing ***high significance (p < 0.001), while RNH2 showed *low significance (p < 0.05). Conversely, R2NH, R3N, RCOOR, ROR, and RCN were observed at significantly higher levels in non-inhibitors, with R2NH, R3N, and ROR showing ***highly significant differences (p < 0.001) and RCOOR and RCN showing **moderate significance (p < 0.01). Minimal or no differences were observed for descriptors like ROPO3, RCHO, and RCCH, which were not statistically significant. These findings suggest that specific functional groups, particularly those involving amines, carbonyls, and aromatic structures, may play important roles in modulating inhibitory activity.

### 3.2 Performance of prediction models

#### 3.2.1 Correlation based descriptor model performance

In this approach, after eliminating highly correlated features, we selected 32 2D, 3 3D and 348 FP descriptors using SVC-L1 based feature selection method. We developed 2D, 3D, FP and ensemble-based machine learning prediction model.

##### 3.2.1.1 2D descriptors-based ML model

Machine learning prediction model was developed using 32 2D descriptors. Using this approach, K-Nearest Neighbor method recorded the maximum AUC for validation set as 0.62 and accuracy as 64.85%, as evident from Table 1. Performance of 2D descriptors over training dataset can be referred in Supplementary Table S4 in Supplementary Material.

**TABLE 1:** The machine-learning model performance on validation dataset developed using 32 2D descriptors.

	Validation							
Model	Accuracy	Precision	Recall	F1	Sens	Spec	AUC	MCC
RF	65.05	0.65	0.49	0.74	0.49	0.56	0.61	0.24
DT	54.23	0.54	0.44	0.63	0.44	0.49	0.54	0.07
KNN	64.85	0.65	0.53	0.72	0.53	0.58	0.62	0.25
SVC	61.33	0.61	0.37	0.77	0.37	0.46	0.57	0.15
XGB	61.33	0.61	0.45	0.72	0.45	0.52	0.58	0.18

##### 3.2.1.2 3D descriptors-based ML model

We have also developed machine learning based model using 3 3D descriptors screened. We observed the maximum AUC over validation set as 0.56 with accuracy as 56.14% in Random Forest classifier, see Table 2. Performance of 3D descriptors over training dataset can be referred in Supplementary Table S4 in Supplementary Material.

**TABLE 2:** The machine-learning model performance on validation dataset developed using 3 3D descriptors.

	Validation							
Model	Accuracy	Precision	Recall	F1	Sens	Spec	AUC	MCC
RF	56.14	0.57	0.49	0.64	0.49	0.52	0.56	0.12
DT	52.31	0.52	0.55	0.50	0.55	0.53	0.52	0.05
KNN	53.52	0.53	0.55	0.52	0.55	0.54	0.54	0.07
SVC	51.51	0.52	0.32	0.71	0.32	0.40	0.51	0.03
XGB	51.91	0.68	0.06	0.97	0.06	0.11	0.52	0.08

##### 3.2.1.3 Fingerprint descriptors-based ML model

In this study, we developed classification models using 348 fingerprints descriptors selected using correlation and SVC-L1 based feature selection approach. As depicted in Table 3, using FP descriptors we achieved a maximum AUC of 0.66 and accuracy as 66.40% over validation dataset using Random Forest classifier. Also, XGBoost model reported the AUC as 0.66, and 65.79% as accuracy for validation set. Performance of FP descriptors over training dataset can be referred in Supplementary Table S4 in Supplementary Material.

**TABLE 3:** The machine-learning model performance on validation dataset developed using 348 FP descriptors**.**

	Validation							
Model	Accuracy	Precision	Recall	F1	Sens	Spec	AUC	MCC
RF	66.40	0.74	0.51	0.82	0.51	0.60	0.66	0.34
DT	57.95	0.59	0.50	0.66	0.50	0.54	0.58	0.16
KNN	65.19	0.66	0.62	0.69	0.62	0.64	0.65	0.30
SVC	59.96	0.61	0.54	0.66	0.54	0.57	0.60	0.20
XGB	65.79	0.68	0.59	0.73	0.59	0.63	0.66	0.32

##### 3.2.1.4 Ensemble based approach

In order to improve the machine learning model performance, we adopted an ensemble-based approach in this study. We combined 32 2D, 3 3D and 348 FP descriptor set selected using SVC-L1 feature selection approach applied after removing highly correlated features. We developed a machine learning model, using a matrix of 383 feature set of 2D, 3D, FP descriptors. As presented in Table 4, we recorded the maximum AUC as 0.67 and accuracy of 67.20% using Random Forest classifier over validation dataset. Performance of FP descriptors over training dataset can be referred in Supplementary Table S4 in Supplementary Material.

**TABLE 4:** The machine-learning model performance on validation dataset developed using 383 ensemble-based descriptors set.

	Validation							
Model	Accuracy	Precision	Recall	F1	Sens	Spec	AUC	MCC
RF	67.20	0.73	0.53	0.81	0.53	0.62	0.67	0.36
DT	56.54	0.57	0.51	0.62	0.51	0.54	0.57	0.13
KNN	64.79	0.66	0.62	0.68	0.62	0.63	0.65	0.30
SVC	60.97	0.62	0.54	0.68	0.54	0.58	0.61	0.22
XGB	64.59	0.68	0.54	0.75	0.54	0.60	0.65	0.30

#### 3.2.2 Univariate analysis-based descriptor model performance

In this approach, we screened top 2,000 descriptors using Univariate analysis. We calculated the mean difference of descriptor score and the single descriptor-based AUC score for top 20 descriptors, refer to Supplementary Table S5 in Supplementary Material. In this, we observed the KRFP605 outperformed all and have shown the maximum AUC as 0.62, with average mean difference as 2.60 among positive and negative data descriptor. In addition to this, we applied SVC-L1 and RFE based feature selection technique over top 2000 descriptors screened using Univariate analysis. We developed machine learning based model for prediction of NF-κB inhibitors using 266 descriptors from SVC-L1 method and the top 50 descriptors from RFE feature selection technique (Refer Supplementary Table S6 in Supplementary Material for detailed list). As depicted in Table 5, Support vector classifier (SVC) developed using SVC-L1 based feature selection technique outperformed all classifiers and reported maximum AUC of 0.80 on training dataset and 0.75 on validation dataset. However, K-nearest neighbor classifier reported maximum AUC of 0.66 on training dataset and 0.65 on validation dataset developed using 50 RFE selected descriptors (See Supplementary Table S7 in Supplementary Material).

**TABLE 5:** The machine-learning models performance on validation dataset developed using 266 descriptors selected using SVC-L1 based approach**.**

	Validation						
Model	Accuracy	Kappa	F1	Sens	Spec	AUC	MCC
RF	65.59	0.31	0.64	60.73	70.40	0.71	0.31
DT	60.36	0.21	0.58	54.25	66.40	0.63	0.21
KNN	65.19	0.30	0.63	60.73	69.60	0.70	0.31
SVC	67.61	0.35	0.66	63.16	72.00	0.75	0.35
XGB	62.78	0.26	0.61	59.51	66.00	0.69	0.26

### 3.3 FDA approved drug repurposing to target NF-κB signaling pathway

In this study, we attempted a systemic approach to identify the potential drug targets for NF-κB signaling pathway. In order to achieve this, we retrieved the 2616 FDA approved drug molecules from Drug Bank portal to screen them as the NF-κB pathway inhibitors and non-inhibitors. Out of 2616, SMILES format was available for 2577 drug molecules. We deployed the “Predict” module of our NfκBIn webserver over these 2577 compounds SMILES format dataset using the default parameters. It computed descriptors using PaDEL software in the backend and provided a machine learning based model label for each drug candidate in 2577 compounds dataset. The machine learning score and predicted label as inhibitor or non-inhibitor for 2577 compounds can be referred in Supplementary Table S8 in Supplementary Material. For top 10 potential drug candidates identified as NF-κB signaling pathway inhibitors, we reviewed previous studies to validate and support our findings. These studies provide evidence of the inhibitory effects of six compounds on the NF-κB pathway, reinforcing the potential of these candidates for further investigation and development (El-Bassoun et al., 2021; Noori et al., 2023; Matthews et al., 1996; Jiang et al., 2019; Sun et al., 2024; Sunaga et al., 2012; Cai et al., 2022). These seven potential drugs, i.e., Bleomycin, Nitroprusside, Guanidine Ivermectin, Tobramycin, Pentosan polysulfate and Gentamicin and their roles as reported in various studies are depicted in Table 6.

**TABLE 6:** List of seven FDA-approved drug candidates as potential Nf-κB pathway inhibitors.

DrugBank ID	FDA approved drugs	Prediction label	Literature remarks
DB00290	Bleomycin	Inhibitor	Co-administration of ginsenoside with BLM resulted in marked improvement in lung structure and a significant reduction in Nf-κB expression. (El-Bassoun et al., 2021)
DB00325	Nitroprusside	Inhibitor	Nitroprusside is a vasodilator that releases nitric oxide (NO) upon metabolism. NO can influence various signalling pathways, including the Nf-κB pathway. (Colasanti and Persichini, 2000)
DB00536	Guanidine	Inhibitor	The guanidine compound ME10092 inhibits Nf-κB activation and the upregulation of inflammatory mediators *in vivo*. (Dambrova et al., 2010)
DB00602	Ivermectin	Inhibitor	Ivermectin may inhibit LPS-induced production of inflammatory cytokines by blocking NF-kB pathway and improve LPS-induced survival in mice. FDA-approved antiparasitic drug, could potentially be used in combination with chemotherapeutic agents to treat cancers. (Noori et al., 2023; Jiang et al., 2019; Zhang et al., 2008)
DB00684	Tobramycin	Inhibitor	Tobramycin suppresses Nf-κB activation, reducing pro-inflammatory cytokines and controlling excessive inflammation in lung infections, thus helping prevent further lung damage in conditions like cystic fibrosis. (Sun et al., 2024; Nguyen et al., 2002)
DB00686	Pentosan Polysulfate	Inhibitor	Pentosan polysulfate sodium (PPS), an inhibitor of NF-kB activation. (Sunaga et al., 2012; Krishnan et al., 2023)

### 3.4 Webserver and standalone package

In this study, we have provided a user-friendly webserver “NFκBin” (https://webs.iiitd.edu.in/raghava/nfkbin/) platform to enable high-throughput screen of chemical compounds as NF-κB inhibitors and non-inhibitors. This webserver is deployed on a Linux (Ubuntu) machine using an Apache HTTP server. Its front-end is created with HTML, PHP, and JavaScript, while the back-end is implemented in Python 3.6 utilizing the Scikit library. In addition to this, to ease the usability of the webserver we have utilized a responsive template which is compatible with desktop, tablet and phone. Major modules incorporated in this webserver, are “Predict,” “Draw,” and “Analog design”. Predict module enables users to screen the chemical compounds in SMILES format as NF-κB inhibitors and non-inhibitors. Best machine learning model has been incorporated in this module with default threshold parameter. Threshold refers to the classification score cutoff used to determine whether a molecule is predicted as an inhibitor or non-inhibitor. Draw module allows users to draw or modify the chemical compound’s structure using an open-source interactive tool known as Ketcher. Post that, drawn structure can be further classified as NF-κB inhibitors and non-inhibitors. In order to generate the analog’s of the chemical compounds using combination of scaffolds, building blocks, and linkers, users can utilize the Analog Design module. SmiLib tool has been implemented in the backend of this module. The tabular format results generated can be downloaded in.csv format from all modules. In addition, we also developed standalone software package which is available from GitHub and PyPI site (https://github.com/raghavagps/nfkbin/& https://pypi.org/project/nfkbin/).

## 4 Discussion

NF-κB is a pivotal therapeutic target due to its dysregulation in chronic inflammation, immune disorders, and cancers. Its activation, particularly through the TNF-α-mediated canonical pathway, leads to nuclear translocation and downstream transcription of pro-inflammatory genes. Researchers have increasingly emphasized the importance of blocking this signaling cascade early to mitigate disease progression. Several tools developed in past focusing on various broader domain such as EGFRpred (Singh et al., 2015) aims to predict the potential chemical molecule as an EGFR inhibitor based on the structure-activity (QSAR model) of the chemical compound; DrugMint (Dhanda et al., 2013) to scan and identify whether a chemical molecule is a potential drug candidate or not; ChAlPred (Sharma et al., 2021) tool for predicting allergenicity of chemical compounds. In addition to these, several molecular docking and simulation-based studies have been conducted for screening of chemical compounds as Nf-kB inhibitors (Saeed et al., 2022; Hua et al., 2020; Kanan et al., 2019; Lo et al., 2017; Leung et al., 2013; Wang et al., 2019; Abbasi et al., 2023; Srivastava et al., 2024). However, tools specifically designed to screen NF-κB pathway inhibitors, especially those targeting the TNF-α axis, remain limited.

While molecular docking and simulation-based methods have been employed for identifying NF-κB inhibitors, they primarily focus on single protein-ligand interactions and often fall short of capturing pathway-level dynamics. These methods are further constrained by their reliance on rigid protein structures and relatively small chemical libraries, leading to limited predictive power and high false-positive rates. In contrast, machine learning approaches—when appropriately trained—can model complex, pathway-level effects by leveraging large datasets of experimentally validated compounds and high-dimensional molecular descriptors. That said, such approaches are not inherently pathway-specific but instead learn associations from the data used.

In this context, we developed NFκBIn, a machine learning-based framework for screening small molecules as NF-κB pathway inhibitors or non-inhibitors, specifically focusing on TNF-α-induced activation. Our dataset comprised 2481 curated compounds (1149 inhibitors and 1332 non-inhibitors), for which we generated comprehensive 2D, 3D, and fingerprint-based descriptors. The Support Vector Classifier (SVC) model developed using the SVC-L1-selected features achieved the best performance, with an AUC of 0.80 on the training dataset and 0.75 on the independent validation dataset. This best-performing model was subsequently implemented as the core prediction engine in our webserver tool for screening NF-κB pathway inhibitors. To support model interpretability, we examined the range of key molecular descriptors in the training dataset. These include ALogP (−4.53–5.03), TPSA (0.00–372.50), molecular weight (173.08–900.44), H-bond acceptors (0–19), donors (0–8), and rotatable bonds (0–15). All new compounds would be processed using the same descriptor generation tool (PaDEL) and Min-Max scaling as the training data. This would enhance the tool applicability and ensure consistent feature representation across datasets.

To demonstrate the utility of NFκBIn in drug repurposing, we screened 2577 FDA-approved drugs from DrugBank. The model predicted several high-confidence inhibitors. Of these, seven compounds—including Bleomycin, Ivermectin, Tobramycin, and Pentosan polysulfate—were supported by literature evidence for modulating NF-κB signaling. In addition, the tool identified other highly ranked compounds with no prior association with NF-κB inhibition. These represent novel candidates for experimental validation and may offer potential for repositioning as anti-inflammatory or anticancer agents.

Although our models showed good performance, we acknowledge certain limitations. For instance, the SVC model displayed imbalanced sensitivity and specificity on validation data, possibly reflecting chemical or assay biases in the training set. Future enhancements may include integrating multi-target or multi-omics features, applying advanced optimization techniques such as genetic algorithms, and validating predictions through biological experiments or docking simulations.

In summary, NFκBIn offers a scalable, interpretable, and user-friendly platform for identifying potential inhibitors of TNF-α-induced NF-κB signaling. It serves as a valuable resource for researchers aiming to accelerate drug discovery and repurposing in inflammatory and cancer-related diseases.

## 5 Conclusion

NF-κBIn method can be implied in Computational drug discovery pipelines to conduct virtual screening of chemical compound libraries as NF-κB inhibitors. Repurposing of FDA-approved drugs as potential candidates against the NF-κB pathway opens new avenues for therapeutic interventions. These findings strengthen the case for further exploration and development of six compounds as viable drug candidates. In addition to this, webserver enable scientific community to create or modify chemical compounds for the discovery of novel chemical compounds targeting against NF-κB signaling pathway.

## Data Availability

All the datasets generated for this study are available at the “NFκBIn” web server, https://webs.iiitd.edu.in/raghava/nfkbin/dataset.php.

## Glossary

NF-κB: Nuclear Factor kappa B
TNF: Tumor Necrosis Factor
FP: Fingerprint descriptor
RFE: Recursive Feature Elimination
MCC: Matthews Correlation Coefficient
DT: Decision Tree
RF: Random Forest
LR: Logistic Regression
XGB: Extreme Gradient Boosting
KNN: K-nearest Neighbour
SVC: Support Vector Classifier
Sens: Sensitivity
Spec: Specificity
Acc: Accuracy
AUC: Area Under the Curve
TLRs: Toll-like Receptors
IL-1: Interleukin-1
IκB: Inhibitor of kappa B
BAFF: B-cell Activating Factor
DMSO: Dimethyl sulfoxide
HEK-293-T: Human Embryonic Kidney 293T Cells
SMILES: Simplified Molecular Input Line Entry System
SVC-L1: Support Vector Classifier with L1 Regularization
PaDEL: Pharmaceutical Data Exploration Laboratory
ALogP: Atom-based Log Partition Coefficient
TPSA: Topological Polar Surface Area
QSAR: Quantitative Structure–Activity Relationship
EGFR: Epidermal Growth Factor Receptor
RNH2: Primary Amines
R2NH: Secondary Amines
R3N: Tertiary Amines
ROPO3: Phosphates attached to alkyl groups
ROH: Alcohol Functional Group
RCHO: Aldehyde Functional Group
RCOR: Ketone Functional Group
RCOOH: Carboxylic Acid Functional Group
RCOOR: Ester Functional Group
ROR: Ether Functional Group
RCCH: Alkyne Functional Group
RCN: Nitrile Functional Group
